# Reproducibility of 3-dimensional ultrasound readings of volume of carotid atherosclerotic plaque

**DOI:** 10.1186/1476-7120-6-42

**Published:** 2008-08-26

**Authors:** Malte Ludwig, Tomasz Zielinski, Dieter Schremmer, Klaus O Stumpe

**Affiliations:** 1 Centre for Vascular Disease, Krankenhaus Tutzing, D-82327 Tutzing, Bahnhofstrasse 5, Germany; 2 Department of Heart Failure and Transplantation, Institute of Cardiology, 04-628, Warsaw, Ul. Alpejska 42, Poland; 3 GKM Gesellschaft für Therapieforschung GmbH, Lessingstr. 14, 80336, Munich, Germany; 4 Department of Hypertension and Cardiovascular Research, University of Bonn, Centre of Preventive Medicine, Herwarthstraße 36, D-53115, Bonn, Germany

## Abstract

**Background:**

Non-invasive 3-dimensional (3D) ultrasound (US) has emerged as the predominant approach for evaluating the progression of carotid atherosclerosis and its response to treatment. The aim of this study was to investigate the quality of a central reading procedure concerning plaque volume (PV), measured by 3D US in a multinational US trial.

**Methods:**

Two data sets of 45 and 60 3D US patient images of plaques (mean PV, 71.8 and 39.8 μl, respectively) were used. PV was assessed by means of manual planimetry. The intraclass correlation coefficient (ICC) was applied to determine reader variabilities. The repeatability coefficient (RC) and the coefficient of variation (CV) were used to investigate the effect of number of slices (S) in manual planimetry and plaque size on measurement variability.

**Results:**

Intra-reader variability was small as reflected by ICCs of 0.985, 0.967 and 0.969 for 3 appointed readers. The ICC value generated between the 3 readers was 0.964, indicating that inter-reader variability was small, too. Subgroup analyses showed that both intra- and inter-reader variabilities were lower for larger than for smaller plaques. Mean CVs were similar for the 5S- and 10S-methods with a RC of 4.7 μl. The RC between both methods as well as the CVs were comparatively lower for larger plaques.

**Conclusion:**

By implementing standardised central 3D US reading protocols and strict quality control procedures highly reliable ultrasonic re-readings of plaque images can be achieved in large multicentre trials.

## Introduction

Measurement of carotid atherosclerosis burden and progression is an important tool for research and patient management [1]. 2-dimensional (2D) B-mode ultrasound (US) has been shown to be a sensitive and reproducible method to detect pre intrusive thickening of artery walls and to measure intima-media thickness (IMT) in the carotid artery [2,3]. Ultrasonographically determined IMT has been used as a marker of atherosclerosis elsewhere in the arterial system and randomised imaging studies have examined the effects of blood pressure- and lipid-lowering therapies on carotid IMT changes [4]. The atherogenic nature of IMT, however, is uncertain, since 2D US does not measure medial and intimal thickness separately [5]. An increase in IMT may be the result of an adapted response of medial layer (remodelling) to tensile (hypertensive) stress or intimal thickening reflecting early atherosclerosis [6]. Atherosclerotic plaque volume assessed by 3-dimensional (3D) US [7] may represent a more reliable measure of atherosclerosis than IMT [8] and more recently non-invasive 3D US imaging has ermerged as the predominant approach for evaluating the progression of carotid atherosclerosis [1,9-12]. 3D US provides a precise and reproducible method for determining the change in atherosclerotic plaque volume during treatment [1,8,10]. A recent randomised trial in hypertensive patients using carotid 3D US has successfully investigated the effects of two different classes of antihypertensive agents on plaque volume changes and demonstrated the suitability of the 3D US method for tracking progression or regression of plaque volume over time [13].

3D US measurement of PV within a multicentre clinical trial setting typically involves processing of data obtained at more than one investigational site. It is important to ensure that analysis of the data, and re-reading of the 3D US images of plaque is carried out in a centralised, standardised and reproducible manner. The primary aim of this study was to assess reliability of a central re-reading procedure implemented in a multinational 3D US trial by determining intra- and inter-reader variabilities and major factors influencing reading precision.

## Materials and methods

The study was performed in accordance with the principles of the Declaration of Helsinki, and the regulatory requirements of the International Conference on Harmonisation Guidelines on Good Clinical Practice.

The protocol was approved by the appropriate Institutional Review Board or Ethics Committee at each centre involved.

All patients gave written informed consent for publication of this report and accompanying images.

### Patients

Two data sets of 45 and 60 3D US carotid plaque images were obtained from 45 patients (32 men; mean [SD] age, 62.2 [6.9] years) and 60 patients (50 men; mean [SD] age, 61.3 [8.1] years), respectively, who had been followed up in the ACAT Inhibition Plaque Regression Study (APRES). The APRES was a multicentre European ultrasound trial, and participants were recruited at 31 clinical centers throughout the Czech Republic, Germany, Italy and Poland. The study assessed the effect of an ACAT-inhibitor as compared to placebo on carotid plaque volume (Data on file at Daiichi Sankyo, Munich, Germany). To be eligible for inclusion in the APRES, patients with defined cardiovascular risk had to have an increased common carotid artery (CCA) intima-media thickness (IMT) of > 0.8 mm, and at least one atherosclerotic plaque in the CCA or the carotid bulb without marked mineralisation (plaque volume between 20 and 500 μl).

### Ultrasound measurement of plaque volume (PV)

Ultrasound measurements of PV had been carried out by trained and certified sonographers at 14 ultrasound referral centers using a high-resolution Voluson 530 D MT, 2D-/3D CFM-Ultrasound System (Kretz-Technik AG, Zipf, Austria; *the equipment is commercially available from General Electric, GE Ultraschall Deutschland GmbH/D-42655 Solingen and named Logiq 7 BT07*) equipped with a mechanical 10 MHz sector motor-driven 3D-probe that provided an axial resolution of 0.1 mm. An integrated 6.9 MHz Doppler system was used to determine the grade of possible stenoses.

**Figure 1:**
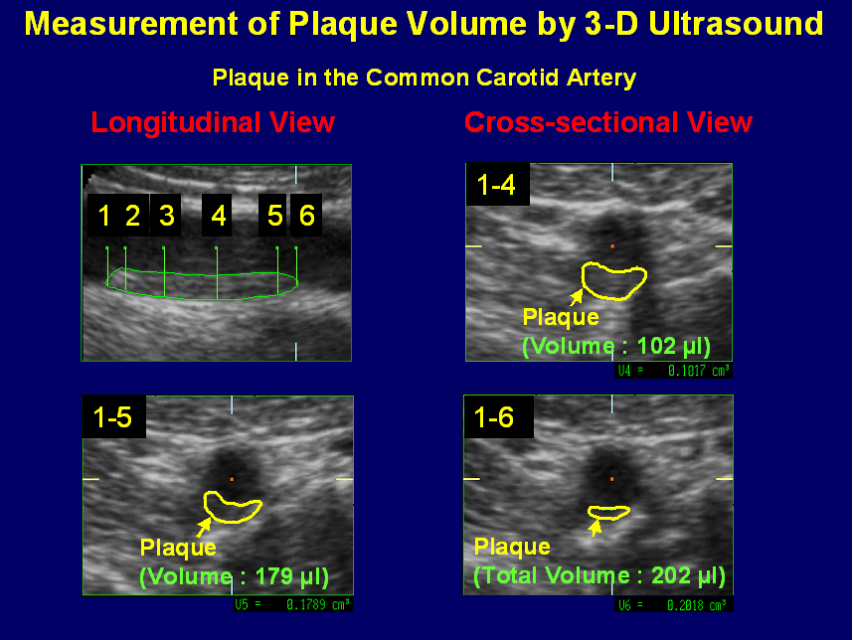
**Carotid plaque images obtained with 3D US.** The plaque is presented in longitudinal and cross-sectional views. The 3D image has been sliced 6 times from one end to the other along the vessel axis in the scan direction.

Small depth of Sweep Box for 3D image acquisition with an angle of 45° and slow acquisition time of volume scan were additional features of the ultrasound system. To measure PV, a longitudinal scan of the common carotid artery or bulb was carried out followed by volume acquisition. Volume acquisition used 3 orthogonal sectional planes (A, B, C) and started with the sectional image (A), which gave a 2D image showing the longitudinal view of the vessel. Images B and C showed the transverse and horizontal axes, respectively. The volume sweep was saved as raw data and stored on MODs and sent to the Reading Center for volume calculations. The volume of analysed plaque was calculated by delineating the plaque boundaries and the transverse plane (B) using the firmware of Kretz Ultrasound apparates. This "manual" method required the object to be manually traced by using the track ball of the Kretz device. In order to achieve volume measurement a manual trace was displayed on different planes of the plaque (Figure 1). The methodology for PV measurement has been validated previously [14-16].

### Quality assessment of central reading

The first set of data consisted of 45 3D US plaque images with PV ranging from 21 to 240 μl. This data set was used to compare the results of plaque volume measurements obtained by 3 trained and certified readers at the European Ultrasound Teaching and Reading Center (EUTARC; Feldafing, Germany) and to investigate intra- and inter-reader variability of the reading process. The second data set consisted of 60 plaque images (PV between 20 and 60 μl) and was used to explore the effect of plaque size and the number of slices (S) (5 vs. 10 S) in manual planimetry on the quality of the central reading procedure by a randomly assigned reader.

### Study protocol and statistical analysis

Sonographic images of the 45 plaques of the first data set were recorded on video tapes and MODs, and 6 identical blinded copies were prepared by an independent institution (Medizinische Software GbR [MESO], Mittweida, Germany) and named A to F. Three sets of the 45 MODs were then sent to the Reading Center EUTARC and randomly assigned to 3 appointed readers for subsequent evaluation. After the measurements from the first round of reading procedure had been returned to MESO, the remaining 3 sets of MODs were delivered to the 3 readers for a second round of readings. Thus, each reader performed two independent evaluations of the 45 MODs according to a randomized, pre-defined procedure. The mean of 3 individual readings was used for analysis.

Inter-reader variability was evaluated by comparing the mean of the first and the second measurement of each reader between all 3 readers. Assessment of intra-reader variability was based on comparison between the first and the second set of measurements of each reader. The intraclass correlation coefficient (ICC) was applied to assess inter- and intra-reader variabilities [17]. The ICC is a measure of the proportion of variance that is attributable to the objects of measurements, and has emerged as a universal and widely accepted reliability index [18]. Furthermore, it allows the evaluation of the variability between more than 2 methods and readers, respectively. Calculation of the ICC was carried out by means of analysis of covariance (ANCOVA) using the SAS program (version 9.1). The three ICC values between the first and second set of measurements of each reader provide an estimation of intra-reader variability, and the ICC between all 3 readers is a measure of inter-reader variability. An additional analysis was performed after stratification by plaque size (PV < 60 μl versus PV > 60 μl) as determined in the APRES.

The second data set was used to investigate the effect of the number of slices in manual planimetry on the quality of PV measurement. The results of the 5 S- and 10 S- method were compared by means of the repeatability coefficient (RC) based on the mean of 3 individual readings per plaque. The RC was calculated as follows [19]: RC = 1.96 SD_Diff_, where SD_Diff _denotes the standard deviation of the differences in PV measurements between both methods. If the differences follow a normal distribution, approximately 5 percent of differences between measurements are expected to lie outside the limits + RC. Variability of measurements was investigated by calculating the coefficient of variation (CV) for 3 individual assessments of each of the 60 plaques by either method. In order to analyse the effect of plaque size (PV) on the precision of measurement, CVs and RC were calculated separately for smaller plaques (PV between 20 and < 40 μl) and larger plaques (PV between 40 and 60 μl).

## Results

### Intra- and inter-reader variability

The results of the PV measurements obtained by the 3 readers for 45 plaques (first data set) are summarised in Table 1. Mean PV values ranged between 67.8 and 71.8 μl. The ICC for intra-reader variability were close to 1 (the highest possible value) for each of the 3 readers with values of 0.985, 0.967 and 0.969 for the first, second and third reader, respectively. The ICC value generated between the 3 readers was 0.964 indicating that inter-reader variability was small, too (Table 1). Intra- and inter-reader variabilities were smaller, i.e. ICCs were higher, for plaques with PV > 60 μl than for plaques with PV < 60 μl (Table 2).

**Table 1:** Inter- and intra-reader variabilities for re-reading plaque volume (PV) of 45 plaques

	**Reader 1**	**Reader 2**	**Reader 3**
**Intra-reader variability**			
PV reading 1 (μl)	71.8 (42.5)	67.8 (42.8)	70.1 (45.4)
PV reading 2 (μl)	71.6 (43.3)	70.0 (43.0)	71.5 (44.0)
ICC	0.985	0.967	0.969

**Inter-reader variability**			
PV both readings (μl)	71.7 (42.7)	68.9 (42.2)	70.8 (44.4)
ICC		0.964	

Values are mean (standard deviation); ICC, intra-class correlation coefficient

**Table 2:** Intra-class correlation coefficients for re-reading plaque volume (PV) of 21 plaques with PV < 60 μl and 24 plaques with PV ≥ 60 μl

	**Reader 1**	**Reader 2**	**Reader 3**
**PV < 60 μl**			
**Intra-reader variability**	0.929	0.783	0.689
**Inter-reader variability**		0.805	

**PV ≥ 60 μl**			
**Intra-reader variability**	0.976	0.960	0.960
**Inter-reader variability**		0.949	

### Effect of number of slices

**Figure 2:**
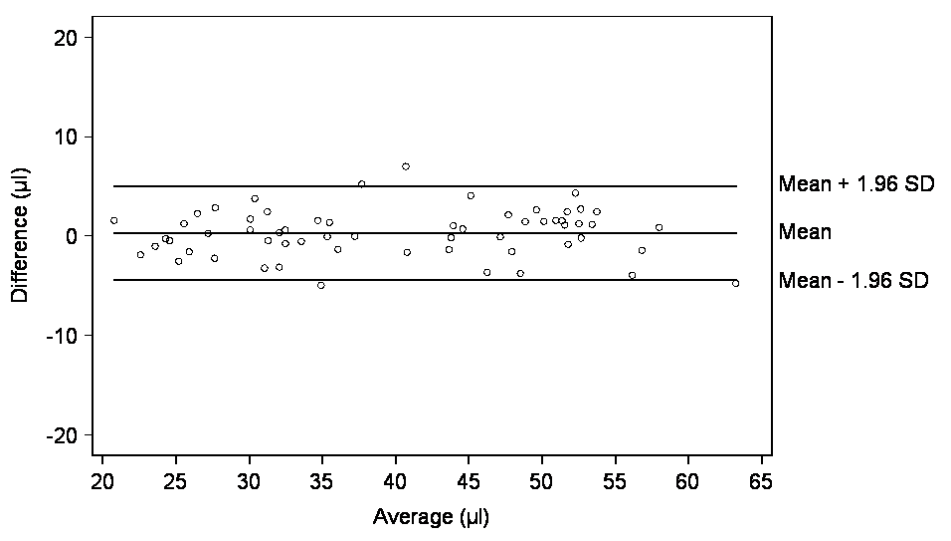
**Difference against average of PV measurements using the 5S- and 10S-methods (second data set, n = 60)**.

Mean (SD) PV of the 60 plaques included in the second data set was 39.8 (11.3) μl for the 5 S-method and 40.1 (11.2) μl for the 10 S-method. Mean (SD) CV calculated from the 3 individual PV measurements per plaque were 3.4 (1.9)% and 3.1 (1.6)% for the 5 S- and 10 S-methods, respectively. The RC was 4.7 μl (Table 3). The corresponding differences in PV measurements between the two methods are depicted by means of a Bland-Altman plot in Figure 2. A stratified analysis of the 30 plaques with PV between 20 and < 40 μl and for the 30 plaques between 40 and 60 μl showed that the mean CVs for both the 5 S- and the 10 S-methods were lower for larger plaques (2.4% and 2.4%) than for smaller plaques (4.3% and 3.8%).

**Table 3:** Coefficients of variation (CV) and repeatability coefficients (RC) for 60 plaques comparing the 5S- and 10S- methods

**PV (μL)**	**N**	**CV (%)**		**RC (μl)**
		
		**5S method**	**10S method**	**5S vs. 10S**
**20 to < 40**	30	4.3 (2.0)	3.8 (1.9)	3.9
**40 to < 60**	30	2.4 (1.1)	2.4 (0.9)	5.3

**20 to < 60**	60	3.4 (1.9)	3.1 (1.6)	4.7

PV, plaque volume

CV was calculated from 3 individual measurements, values are mean (standard deviation)

RC was calculated for mean of 3 individual measurements comparing 5S and 10S

## Discussion

Because carotid plaque progression is not limited to changes in one direction, it is important to measure progression in three dimensions. Plaques grow and regress circumferentially as well as in length and thickness. As a non-invasive technique, 3D imaging allows:

- direct plaque visualization

- quantification of plaque features

- the possibility of investigating volume changes that occur in multiple dimensions, such as plaque surface morphology, plaque geometry, and plaque distribution.

For these reasons 3D-ultrasound is becoming more important in serial monitoring of disease progression or regression. Sample sizes which are required to test the effects of new therapies might be smaller for measurements of plaque volume than for traditional 2D measurements.

The present study aimed to assess the quality of a centralised reading procedure of 3D US recordings of carotid PV measured in a multinational clinical trial. The small intra- and inter-reader variabilities reported in the study validate the reproducibility and reliability of the centralised PV measurement reading technique. Any slight differences observed between the 3 appointed readers may have reflected a minor subjective element due to differences in practice; however, these differences were small and randomly distributed, and would not be expected to have a significant effect on results generated in a clinical trial setting. Individual deviations between the first and second measurement made by the same reader, and between participating readers, were comparable with the occurrence of outliers in other methods of measurement applied in clinical trials, with respect to magnitude and frequency.

In the present study, variability of the re-reading procedure was dependent on plaque size. Both inter- and intra-reader variabilities were lower for larger than for smaller plaques. This finding is in agreement with previous PV variability studies which utilised 3D ultrasound [1,14,20,21] showing that the CV in the measurement of PV decreased with plaque size. Due to irregular shape of some of the atheromatic plaques an increase in the number of slices used for calculation in manual planimetry method by decreasing the distance between slices could possibly increase the accuracy of volume determinations. Therefore, in the present study additional analyses were undertaken on a sample of 60 plaques to assess the variability depending on plaque size and the number of slices used during the determination of plaque volume. Half of plaques were between 20 and < 40 μl and half were between 40 and < 60 μl. The results indicate that the 5S- and the 10S-method provided similar results in volume calculation. Subgroup analyses of the two tracing techniques demonstrated that for the smaller plaques (PV between 20 and < 40 μl), the 10S-method offered slightly greater reliability as indicated by a lower coefficient of variation. In contrast, there was no difference between reliability of the 5S- or 10S-method in the larger plaque group (PV between 40 and < 60 μl). Therefore, the reliability of measurements of smaller plaques (< 40 μl) may be improved by using the 10S-method.

The small variability in 3D US PV measurements and re-readings is an important finding, given the fact that this approach may be useful for evaluating the progression of carotid atherosclerosis and its response to treatment and for targeting preventive therapy [12]. Poor reproducibility could lead to inappropriate clinical management of individual patients, particularly given that the ratio of random measurement error to the variability among progression rates is large, and repeated measures or longer follow-up would be required to sufficiently reduce the contributions of random error to allow individual diagnoses [22].

**Figure 3:**
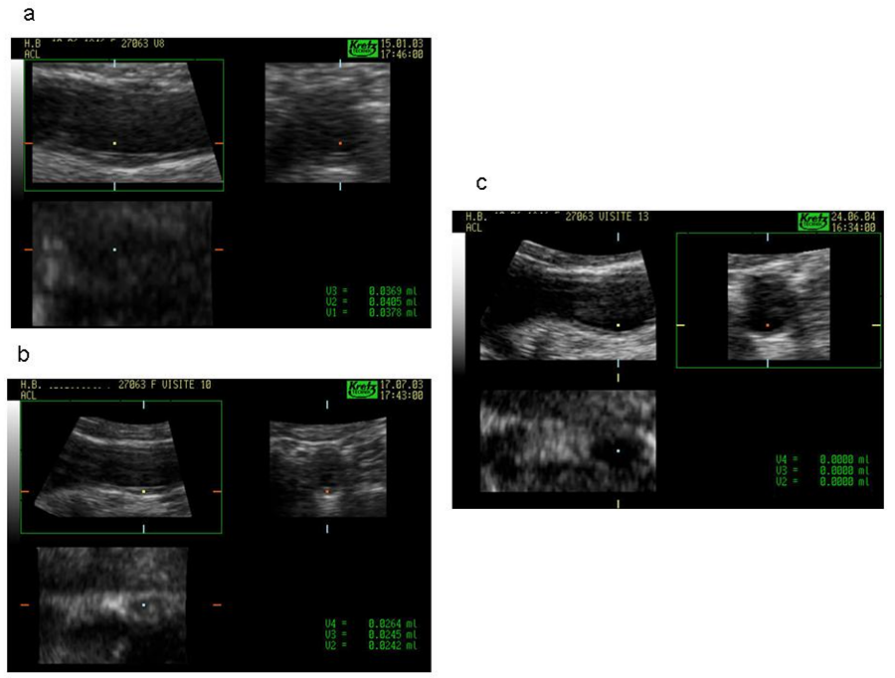
**Carotid plaque images obtained with 3D US showing an example of plaque regression under therapy with a daily dose of 40 mg olmesartan:** a) A fibrous non-calcified plaque of the carotid artery (bulb) presented in longitudinal and cross sectional views at the start of therapy (Mean volume: 384 μL). b) The same plaque during therapy six months later (Mean volume: 250 μL). c) The same plaque (no longer visible) 16 months later.

In contrast to disease areas such as cancer, large-scale screening programs to identify at-risk individuals with atherosclerotic disease have not yet been introduced, despite the higher burden of associated morbidity and mortality, as highlighted by the recent SHAPE Task Force Report [23]. The present study has confirmed that a reliable, non-invasive technique for monitoring the progression or regression (Fig. 3) of carotid atherosclerosis is now available.

It should be noted that a number of limitations can affect the 3D technique for measuring plaque volume. In a small number of cases, plaque boundaries might not be well defined due to dropouts and shadowing owing to attenuation of the US beam, and these might be present in the reconstructed 3D US images. Calcified plaques with marked shadowing are not measureable, and plaque identification at the carotid bifurcation and in areas of poor image resolution may in a few cases also create some difficulty in plaque identification. In addition, there is a higher variability in smaller plaques (< 40 μl), and the reliability of measurements of smaller plaques (< 40 μl) may be improved by using an increased number of slices (10 instead of 5 slices).

In summary, the centralized reading procedure investigated in this study has been shown to be reliable and reproducible. Variability in the reading process increases with decreasing plaque volumes, and the 10S-method may offer greater reproducibility than the 5S-method for volume assessment of small plaques.

## Conclusion

The 3D US techniques combined with a well controlled centralised reading procedure described in this study are appropriate tools for monitoring progression of carotid atherosclerosis and its response to treatment. By implementing standardised central 3D US reading protocols and strict quality control procedures highly reliable ultrasonic re-readings of plaques images can be achieved in large multicentre trials.

## Competing interests

Prof. Dr. M. Ludwig – consultant for Daiichi Sankyo, Munich, Germany.

Prof. Dr. K.O. Stumpe has received research grants and lecture honoraria from Daiichi-Sankyo during the last five years.

Prof. Dr. Tomasz Zielinski – consultant for Daiichi Sankyo, Munich, Germany.

## Authors' contributions

Carotid artery scans were carried out by TZ. Reading of scans was carried out by the reading centre EUTARC/Feldafing, Germany. Statistical analyses had been carried out by DS. ML and TZ provided expert input during the analysis and interpretation of the data. All authors provided input during the writing and editing of the manuscript.
